# Enhancing equity and coverage through supply- and demand-side integration policies for maternal/newborn health: Field experience from rural Western China

**DOI:** 10.1016/j.lanwpc.2021.100112

**Published:** 2021-03-01

**Authors:** Erin Anastasi, Willibald Zeck, Sainan Zhang

**Affiliations:** a UNFPA, Campaign to End Fistula, New York, USA; b UNFPA, New York, USA

Ending preventable maternal and newborn deaths and disabilities remains a key global priority, central to achieving a healthier, more just and equitable world, as highlighted in the Sustainable Development Goals [1]. To achieve this, increased effective and cost-effective efforts are needed to expand and improve access, coverage and quality of health care (including primary health care services), with a view toward achieving universal health coverage of key life-saving and essential health services.

Over two million newborns die each year and newborn deaths account for 47% of deaths among children under five globally. Most of these deaths are preventable [2]. Over the past decade, newborn survival and health has been increasingly recognized as a critical area of maternal/child health. Significant breakthroughs resulting from increased investment, evidence, and political commitment have yielded gains in newborn survival and health worldwide. Yet, far more is needed, as called for in the *Every Newborn Action Plan (ENAP)* [3], a global road map for ending preventable newborn and maternal mortality and stillbirth, endorsed by 194 Member States at the World Health Assembly in 2014 (Resolution WHA 67.10).

High quality health systems could save over eight million lives per year in low- and middle-income countries (LMIC) [4]. Increasingly, the global community is recognizing that measuring and ensuring survival alone is not enough. Individual health, well-being and quality of life – or “thriving” – is equally essential, as clearly emphasized in the *Global Strategy for Women's, Children's and Adolescents’ Health* (2016 – 2030) [5].

Antenatal care represents an important opportunity for reaching women, preventing, diagnosing and/or treating maternal and perinatal conditions and, potentially, for improving health outcomes [6]. This includes congenital anomalies. Congenital anomalies or birth defects, defined as structural or functional anomalies that occur during intrauterine life, are one of the main causes of the global burden of disease, and low- and middle-income countries are disproportionately affected. Approximately 50% of congenital anomalies cannot be linked to a specific cause and an estimated 6% of babies worldwide are born with a congenital anomaly, resulting in hundreds of thousands of associated deaths [7]. Several birth defects can be prevented by increasing services for treatment and detection, including strengthening antenatal care services. Recognizing that prevention and care services play an important role, WHO and partners have recently launched a new edition of the birth defects surveillance toolkit [8].

Ensuring progress in maternal and newborn health and well-being necessitates addressing both supply-side and demand-side barriers and facilitators. Essential and life-saving services need to be available, accessible, acceptable and of high quality [9]. In addition, to help women/families/individuals overcome obstacles such as poverty, distance, socio-cultural barriers, etc., and to promote equity and reach those hardest to reach, interventions such as demand-side financing (e.g., vouchers, conditional and unconditional cash transfers, etc.) are increasingly being piloted and implemented in a number of LMICs.

By evaluating a pilot program designed to integrate prenatal screening interventions for congenital abnormalities into routine antenatal care in western rural China, Xin Ling Feng and Chunmei Wen [10] assessed the effects of such screening programs on coverage and equity by comparing four counties within Shaanxi Province and two counties in Ningxia Province, China. The study sought to identify effective policy solutions to promote accessibility and quality of the prenatal screening in routine antenatal care within the *National Essential Public Health Program*.

With congenital anomalies contributing to 10.8% of China's burden of neonatal mortality, the results of this study, published in *The Lancet Regional Health – Western Pacific*, provide valuable evidence from the clinical research perspective on the effectiveness of China's two interventions: providing free prenatal foetal aneuploidies screening and prenatal B-ultrasound screening. Findings from this research support the country's efforts of integrating screening for congenital abnormalities into routine antenatal care, and contributes to the *National Essential Public Health Program*, which is currently rolling out throughout the country.

Results of the research indicate that the coverage of prenatal fetal aneuploidies and B-ultrasound screening rose by over 20 percent. “Supply-side policies” appeared to be more effective for women with secondary education and above, while “demand-side policies” appeared more effective for women with primary education and below and those from remote mountainous areas.

The study involved some limitations. Causal conclusions cannot be made, due to the study design (observational) as well as potential sources of bias (e.g., selection bias due to non-random selection of counties, recall bias), although adjustment was made for demographic and socio-economic factors, to reduce bias. Additionally, the research examined topology/geography (e.g., mountainous versus plains regions); however, other factors could be relevant to women's access to antenatal care services, such as public transportation availability and road conditions. Furthermore, Ningxia is an autonomous region inhabited by Hui minority groups. Culture and ethnicity issues could potentially impact service coverage when comparing the counties. Furthermore, the research would be strengthened if discussion on the coverage rate of prenatal care for the disadvantaged population (e.g. “floating population”) were included, together with recommendations on policies and programs. Finally, the study did not assess the health impact of this initiative.

Nevertheless, this study makes an important contribution by bridging clinical medicine with policies and interventions. It contributes to the evidence base on delivering integrated maternal/newborn health services in China. With congenital anomalies contributing to over 10% of neonatal mortality nationally, it addresses an important public health issue in China. The study helps fill the evidence gap regarding coverage and equity of prenatal screening in rural China, as well as the role of demand-side financing. Furthermore, the research offers insight into the variable impacts of interventions on different population groups.

Additional research to assess the impact of such initiatives on newborn and child survival and health in remote/rural/underserved settings similar to those of rural western China, is recommended. Linking the increase of antenatal care and integrating congenital anomaly screening with the effects on newborn's/children's health using temporal collected data would be more powerful to provide evidenced-based results to decision makers and health professionals. Including key questions in the follow-up routine collected data is recommended for future work.

## Declaration of Competing Interest

We declare no competing interests.
